# Edible Fungus Compound Cordycepin Protects Against Acetaminophen‐Induced Liver Injury

**DOI:** 10.1002/jcla.70266

**Published:** 2026-05-25

**Authors:** Chunjin Fu, Shuyu Li, Shengnan Shen, Chengchao Xu, Jingjing Liao, Na Lin

**Affiliations:** ^1^ State Key Laboratory for Quality Assurance and Sustainable Use of Dao‐di Herbs China Academy of Chinese Medical Sciences Beijing China

**Keywords:** acetaminophen, anti‐apoptotic, anti‐inflammatory, antioxidant, cordycepin, *Cordyceps militaris*, ER stress

## Abstract

**Background:**

Medicinal–edible natural products like *Cordyceps militaris (C.militaris)*, a functional food rich in cordycepin (COR), are promising for health maintenance. Although COR possesses anti‐inflammatory and antioxidant properties, its protective efficacy and underlying mechanisms against acetaminophen (APAP)‐induced hepatotoxicity remain unclear. This study aimed to evaluate the influence of COR pretreatment on APAP‐induced acute liver injury and elucidate the underlying mechanisms.

**Methods:**

To this end, male C57BL/6 mice were gavaged for 7 days with NAC (N‐acetylcysteine) (200 mg/kg) or COR (50, 100, or 200 mg/kg), followed by APAP administration (400 mg/kg, gavage) to cause liver injury for 12 h. Liver injury was evaluated by serum transaminase levels, histopathological analysis, inflammatory cytokine detection, oxidative stress markers, TUNEL staining, and RNA‐seq‐based transcriptomic analysis.

**Results:**

COR pretreatment markedly reduced serum transaminases and alleviated hepatic necrosis and inflammatory infiltration. RNA‐seq revealed significant APAP‐induced changes in genes associated with ER protein processing, HIF‐1, and PPAR signaling, while COR reversed multiple key genes involved in oxidative stress, inflammation, and apoptosis. Consistent with transcriptomic findings, COR decreased pro‐inflammatory cytokines, mitigated oxidative damage, and inhibited hepatocyte apoptosis (TUNEL staining).

**Conclusion:**

In conclusion, COR pretreatment attenuates APAP‐induced hepatotoxicity in mice by modulating ER proteostasis‐related pathways and coordinately influencing HIF‐1 and PPAR‐associated signaling networks, thereby exerting anti‐inflammatory, antioxidant, and anti‐apoptotic effects. These findings support COR as a promising hepatoprotective candidate for APAP‐caused liver damage.

## Graphical abstract

This study demonstrates that pretreatment with cordycepin (COR) effectively protects against acetaminophen (APAP)‐induced acute liver injury in mice. COR mitigates liver damage by exerting strong antioxidant, anti‐inflammatory, and anti‐apoptotic effects through the modulation of ER proteostasis, HIF‐1, and PPAR signaling networks. Ultimately, these findings highlight COR as a promising natural therapeutic candidate for preventing drug‐induced hepatotoxicity.

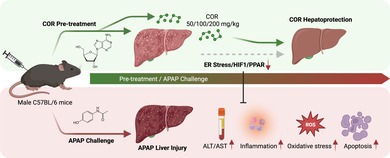

## 1 Introduction

In Traditional Chinese Medicine, the concept of Medicine–food homology (MFH) is rooted in long‐standing traditions of dietary therapy and herbal practice [1]. It refers to natural materials that are consumed as foods while being historically recognized for health‐related functions [2]. As elegantly summarized by Cathie Martin, “Medicine is not health care, food is health care” [3]. Indeed, accumulating evidence supports that bioactive constituents in functional foods hold considerable potential for health promotion and disease prevention. For example, curcumin from turmeric ( *Curcuma longa* ) has been extensively investigated for its anti‐inflammatory and hepatoprotective activities, whereas epigallocatechin gallate (EGCG) from green tea is well‐recognized for its antioxidant benefits in alleviating metabolic and cardiovascular stress [4, 5]. Bioactive constituents from MFH resources are often characterized by multi‐target, modulatory activity patterns, making them attractive as supportive or adjunct approaches that complement—rather than replace—standard therapies [6]. By acting on shared pathological nodes such as inflammation, oxidative stress, and metabolic stress, MFH‐derived molecules may help buffer injury cascades and support recovery, particularly in contexts where a wider safety margin and longer‐term management are desirable [7]. At the same time, the broader “food as medicine” concept has gained increasing international attention, highlighting potential advantages related to accessibility, source traceability, and translational feasibility [8].

**FIGURE 1:**
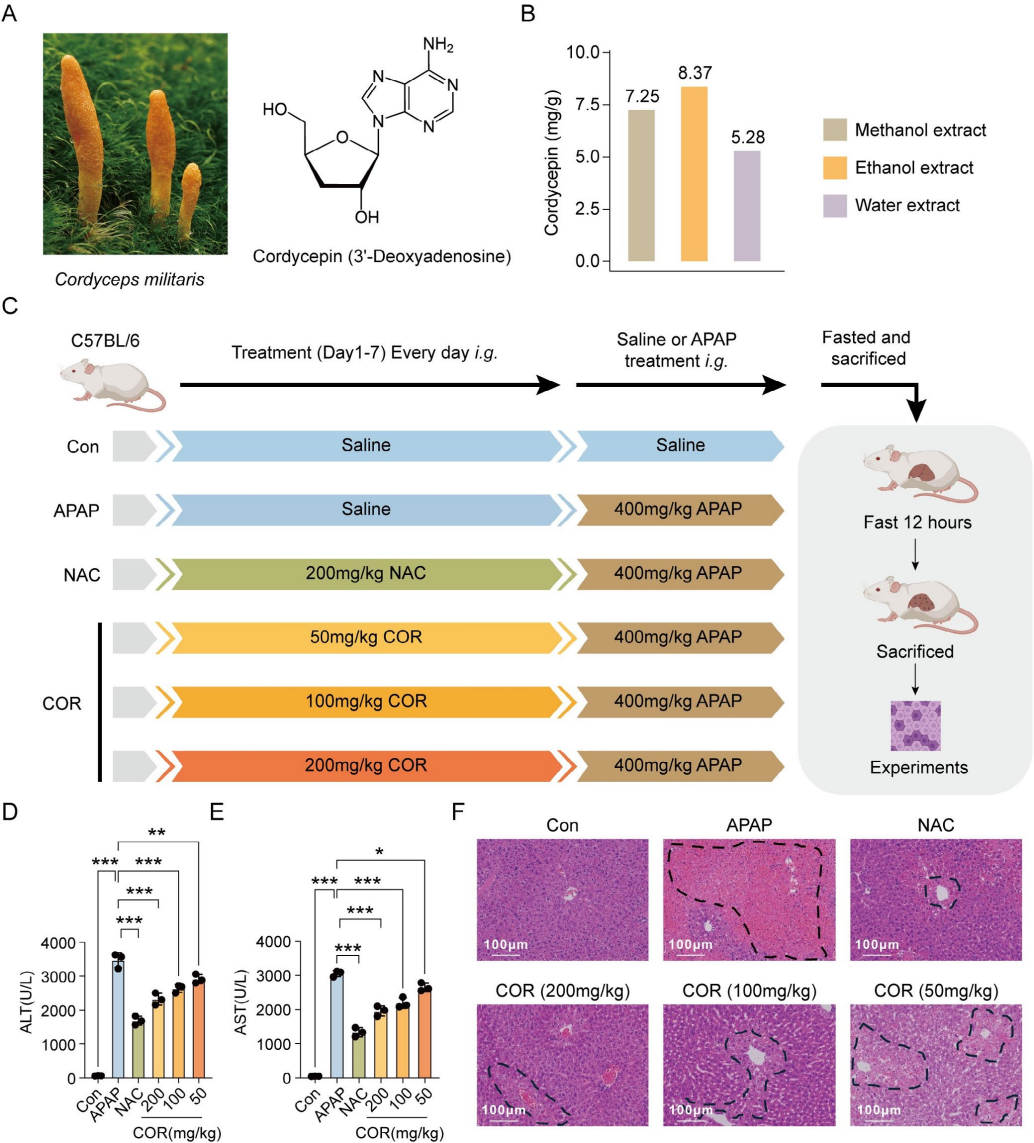
Cordycepin (COR) pretreatment attenuates acetaminophen (APAP)‐induced acute liver injury in mice. (A) Representative image of *Cordyceps militaris* and the chemical structure of cordycepin (3′‐deoxyadenosine), the major bioactive nucleoside derived from this medicinal and edible fungus. (B) Quantification of cordycepin content (mg/g) in different *C. militaris* preparations/extracts [14]. (C) Experimental design. Male C57BL/6 mice were gavaged (*i.g*.) once daily for 7 days with saline (Con and APAP groups), N‐acetylcysteine (NAC, 200 mg/kg), or COR (50, 100, or 200 mg/kg), followed by a single gavage of APAP (400 mg/kg) to induce acute liver injury. Mice were fasted for 12 h after APAP administration and then sacrificed for sample collection and subsequent analyses. (D, E) Serum alanine aminotransferase (ALT, (D)) and aspartate aminotransferase (AST, (E)) activities. Data are presented as mean ± SD (*n* = 3). Statistical analysis was performed using one‐way ANOVA followed by Tukey's multiple‐comparisons test. *p < 0.05, **p < 0.01, ***p < 0.001 (comparisons as indicated). (F) Representative H&E‐stained liver sections showing histopathological changes. Dashed outlines indicate lesion areas. Scale bar, 100 μm.

Medicinal food with a long history of human consumption is increasingly viewed as promising candidates for supportive care and health maintenance, in part because their dietary use, source traceability, and accessibility may facilitate translational exploration alongside purely synthetic small molecules [9]. Among these resources, *Cordyceps* fungi—particularly *C. militaris* —have been widely used in Asia as functional foods and nutraceutical supplements and are rich in diverse bioactive constituents, including cordycepin, ergosterol, polysaccharides, nucleosides, and peptides [10, 11, 12, 13, 14] (Figure 1A). Comprehensive chemical and pharmacological reviews have summarized broad bioactivities of *Cordyceps* preparations, including anti‐inflammatory, antioxidant, immunomodulatory, and anti‐apoptotic effects, together with reported hepato‐ and nephro‐protective potential in experimental settings [10, 15, 16]. COR is a key important nucleoside component in *C. militaris* . Quantitative analysis of COR levels in different *C. militaris* preparations indicated that ethanol and chloroform/methanol extracts contained relatively higher COR contents (8.37 and 7.25 mg/g, respectively) than the water extract (5.28 mg/g) (Figure 1B) [17]. Accumulating studies on *C. militaris* extracts and COR suggest modulatory effects on inflammatory cascades and oxidative stress–related responses—pathological drivers that are central to many forms of tissue injury [18, 19, 20, 21, 22, 23, 24, 25]. These characteristics provide a rationale to further evaluate *C. militaris* –derived molecules, including COR, in clinically relevant contexts where inflammation–oxidative stress–cell death programs converge, such as acute liver injury.

APAP overdose remains a prototypical cause of dose‐dependent drug‐induced acute liver injury and is a leading contributor to acute liver failure in many countries [26, 27]. Mechanistically, APAP is bioactivated by hepatic cytochrome P450 enzymes to the electrophile N‐acetyl‐p‐benzoquinone imine (NAPQI), which depletes glutathione (GSH) and forms protein adducts, thereby promoting mitochondrial dysfunction, excessive reactive oxygen species generation, lipid peroxidation, and downstream hepatocellular death [28, 29, 30]. Beyond the canonical NAPQI–GSH–mitochondrial oxidative stress axis, stress‐response programs (e.g., ER proteostasis), sterile inflammation, and other metabolic adaptations can further shape injury amplification and tissue outcomes, supporting the view that APAP toxicity is a multi‐pathway process [31, 32]. Clinically, NAC remains the primary antidote, acting through glutathione replenishment and improved detoxification [33]; however, the management can be more challenging after massive ingestion [34]. In addition, NAC administration may be complicated by treatment‐related reactions (e.g., anaphylactoid events in a subset of patients), which can necessitate protocol adjustments [35]. Together, these limitations and the complex biology of APAP hepatotoxicity underscore the need for preventive or adjunct strategies that may complement NAC by targeting broader stress–injury networks beyond GSH replenishment alone.

In this study, we established a mouse model of APAP‐caused acute liver injury and applied COR pretreatment with NAC as control to evaluate the hepatoprotective effects of COR. By integrating transcriptomic profiling with biochemical and histopathological validation, we analyzed the effects of COR against such liver injury and provided experimental evidence supporting COR as a potential hepatoprotective candidate.

## 2 Result

### 2.1 COR Attenuates APAP‐Induced Liver Injury

To investigate the prophylactic hepatoprotective effects of COR against APAP‐induced hepatotoxicity, we established an acute liver damage model in male C57BL/6 mice. Male mice were exclusively utilized because they are significantly more susceptible to APAP‐induced hepatotoxicity than females [36, 37]. In this model, mice were pretreated via gavage with NAC (200 mg/kg) or COR (50, 100, or 200 mg/kg), followed by APAP administration to induce liver injury. The mice were sacrificed for analyses 12 h after APAP dosing under fasting conditions (Figure 1C). Compared with the control group, APAP treatment markedly increased serum ALT and AST concentrations (Figure 1D,E), indicating compromised hepatocellular membrane integrity and overt liver injury [38]. Histologically, extensive hepatocellular necrosis or architectural disruption accompanied by inflammatory cell infiltration (dashed areas) was evident in the APAP group, whereas NAC markedly alleviated these pathological changes (Figure 1F). Notably, COR pretreatment also reduced APAP‐induced ALT/AST elevations and improved histopathological damage, with the COR (200 mg/kg) group exhibiting the most pronounced protection (Figure 1D–F). Collectively, these results indicated that COR pretreatment mitigates APAP‐induced acute liver injury at the phenotypic level.

### 2.2 Investigation of COR‐Modulated Signaling Pathways

**FIGURE 2:**
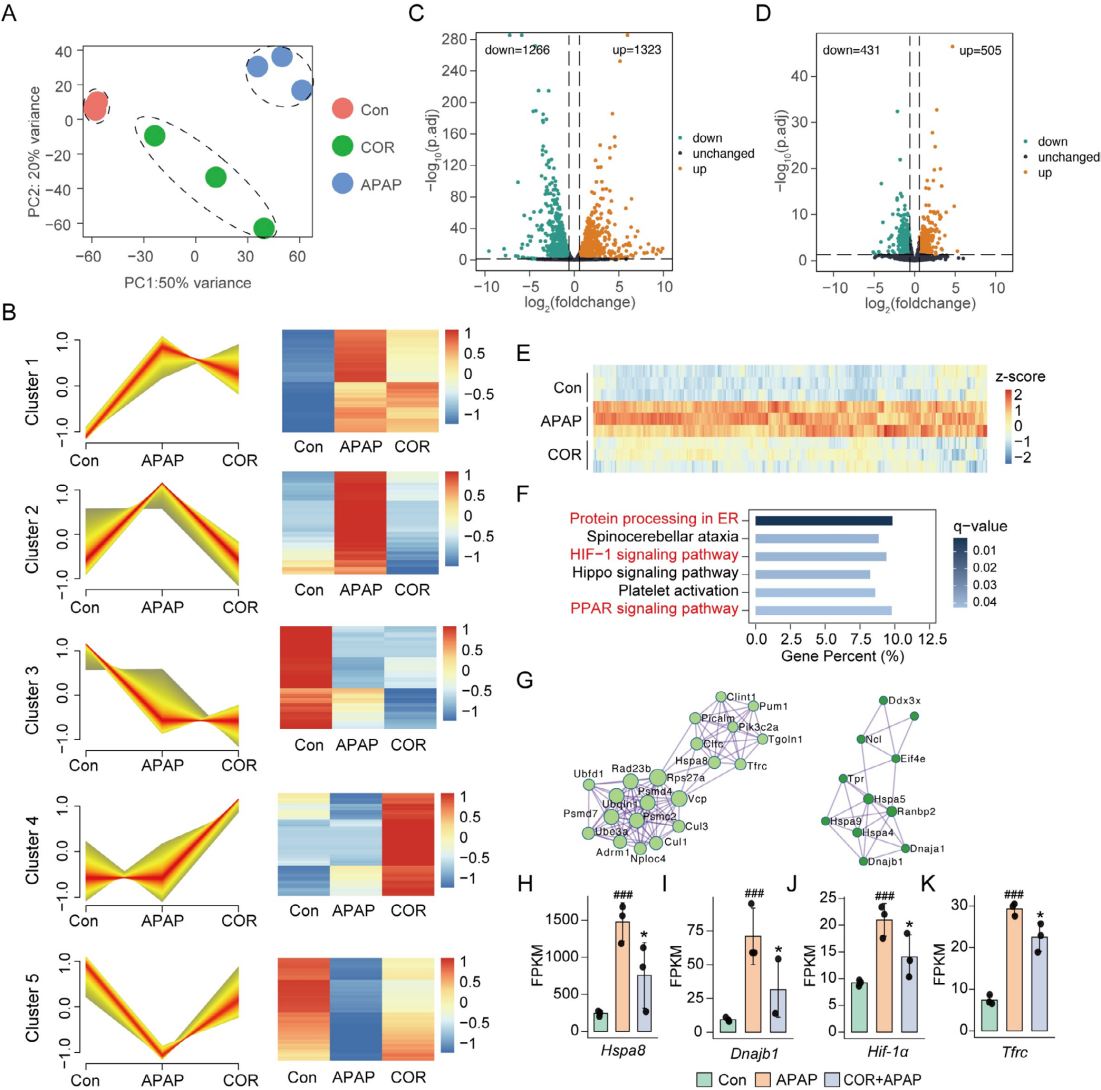
Transcriptome analysis reveals the signaling pathways influenced by cordycepin. (A) Principal component analysis (PCA) of RNA‐seq data from Con, APAP, and COR + APAP (COR 200 mg/kg) groups. (B) Dynamic clustering of genes across the Con, APAP, and COR + APAP (COR 200 mg/kg) groups. Left, average expression trajectories for each cluster; right, corresponding heatmaps showing normalized gene‐expression patterns (z‐score) within each cluster. (C) Volcano plot of DEGs between Con and APAP groups (DESeq2). Vertical dashed lines indicate fold‐change cutoffs and the horizontal dashed line indicates the significance threshold; numbers denote up‐ and downregulated DEGs. (D) Volcano plot of DEGs between APAP and COR + APAP groups (DESeq2), displayed as in (C). (E) Heatmap of the APAP‐upregulated but COR‐reversed genes. (F) KEGG enrichment analysis of the genes in (E). Bar length indicates the percentage of genes mapped to each pathway (Gene Percent), and color denotes the q‐value. (G) Protein–protein interaction (PPI) network of the 217 related COR‐reversed genes, showing highly connected interaction modules. (H–K) RNA‐seq expression levels (FPKM) of representative genes involved in ER proteostasis and stress responses (*Hspa8* (H) and *Dnajb1* (I)), and HIF‐1/iron homeostasis signaling (*Hif‐1α* (J) and *Tfrc* (K)) in Con, APAP, and COR + APAP groups. Data (from DEseq2) are presented as mean ± SD (*n* = 3). ***p < 0.001, **p < 0.01, *p < 0.05 versus APAP group; ###*p* < 0.001 versus control group.

To systematically elucidate the molecular basis underlying the protective effects of COR on the drug‐induced liver injury, we performed RNA‐seq on the COR + APAP (COR 200 mg/kg) group, which exhibited the most robust protection, and compared three groups (Con, APAP, and COR + APAP; Table S2). The PCA results showed clear segregation among the three groups at the global transcriptome level, indicating that APAP induced marked transcriptional remodeling, whereas COR pretreatment substantially reshaped the APAP‐driven expression pattern (Figure 2A).

Clustering of genes based on expression dynamics yielded five distinct clusters (Figure 2B). Notably, subsets of genes were upregulated by APAP but returned toward baseline after COR pretreatment (or vice versa), displaying a “COR‐reversed” expression signature. Differential expression analysis identified 2589 DEGs between Con and APAP groups (Figure 2C) and 936 DEGs between APAP and COR + APAP groups (Figure 2D). Functional enrichment of these DEG sets preliminarily suggested that COR may counteract APAP‐induced liver injury by modulating pathways associated with protein homeostasis (Figure S1A,B). To further pinpoint COR‐responsive targets underlying this protective effect, we focused on the overlap between APAP‐driven and COR‐reversed transcriptional changes. Intersecting the two DEG lists, we extracted 217 candidate genes that were upregulated by APAP but downregulated by COR (Figure 2E), indicating a gene subset most closely associated with COR‐mediated protection.

KEGG enrichment analysis of the 217 COR‐reversed genes revealed significant overrepresentation of pathways related to ER protein processing, HIF‐1 signaling, and PPAR signaling (Figure 2F). The prominence of the “Protein processing in ER” term indicates that APAP challenge imposes a substantial proteostatic burden, likely triggering activation of the chaperone. Consistently, protein–protein interaction (PPI) network analysis showed a highly connected interaction module among proteins encoded by these genes, suggesting coordinated regulation within the stress‐response network (Figure 2G).

HSPA8 (also named HSC70) and DNAJB1 (an Hsp40 family co‐chaperone) constitute key nodes in the protein quality‐control network, participating in recognition and refolding of misfolded proteins and coupling to ER stress and chaperone‐mediated degradation. Previous evidence indicates that APAP hepatotoxicity induces pronounced proteostatic stress and activates heat‐shock programs. While stress‐inducible heat shock proteins are commonly elevated, the constitutive chaperone HSC70/HSPA8 may exhibit variable changes depending on the model; nevertheless, as a core quality‐control factor, the increase of HSPA8 likely reflects APAP‐associated proteostasis disruption [39]. Moreover, toxicoproteomic studies of drug‐induced liver injury have reported higher circulating levels of cytosolic proteins such as HSPA8, suggesting associations with hepatocellular injury and stress responses [40]. Consistent with these observations, our results show that APAP exposure was accompanied by elevated *Hspa8* expression, whereas COR pretreatment attenuated this abnormal upregulation, suggesting that COR may alleviate the proteostatic burden induced by APAP (Figure 2H). In addition, APAP exposure has been shown to rapidly induce chaperone genes like *Dnajb1*, reflecting activation of protein quality‐control and folding responses under ER stress [41, 42]. In line with this, we observed a significant increase in *Dnajb1* expression following APAP challenge, which was markedly reduced by COR administration (Figure 2I). Taken together, these results indicate that COR can mitigate APAP‐triggered ER stress, contributing to a comprehensive restoration of proteostasis.

HIF‐1α is a central transcriptional regulator under hypoxic and oxidative stress conditions, driving diverse inflammatory and metabolic adaptive responses and closely linked to cell‐death outcomes during hepatotoxic stress. Previous studies have shown that APAP overdose robustly induces hepatic HIF‐1α expression at early stages (e.g., ~10‐fold upregulation at 6 h), supporting the notion that APAP‐associated oxidative stress triggers a “hypoxia‐like” transcriptional response [43, 44, 45]. Consistent with these reports, our results revealed a marked increase in *Hif‐1α* expression following APAP administration at the mRNA level. Notably, COR pretreatment substantially attenuated this APAP‐induced elevation (Figure 2J), suggesting that COR may restrain excessive activation of the HIF‐1–dependent stress program and thereby limit downstream inflammatory and metabolic maladaptation.

TFRC (also named TFR1) is a key mediator of cellular iron uptake; its upregulation increases the labile iron pool, thereby promoting Fenton chemistry, amplifying lipid peroxidation, and exacerbating oxidative damage and cell‐death signaling. Consistently, APAP has been reported to markedly increase TFR1 protein levels in mouse liver, and TFR1 is a recognized HIF‐1α target gene through which HIF‐1α modulates cellular iron availability and thereby facilitates hepatocellular ferroptosis [43, 46, 47]. In agreement with this mechanistic framework, we observed significant upregulation of *Tfrc* in the APAP group, whereas COR pretreatment effectively reduced its expression (Figure 2K).

The parallel suppression of *Hif‐1α* and *Tfrc* by COR supports the notion that modulation of the HIF‐1–iron axis may represent an important component of COR‐mediated hepatoprotection, potentially by limiting iron‐driven lipid peroxidation and attenuating ferroptosis‐associated signaling. Collectively, these findings indicate that COR not only mitigates inflammatory and oxidative stress responses but may also recalibrate hypoxia‐like transcriptional adaptation and iron metabolism during APAP‐induced liver injury.

### 2.3 qRT‐PCR and Western Blot Validation of Key Gene‐Expression Changes

**FIGURE 3:**
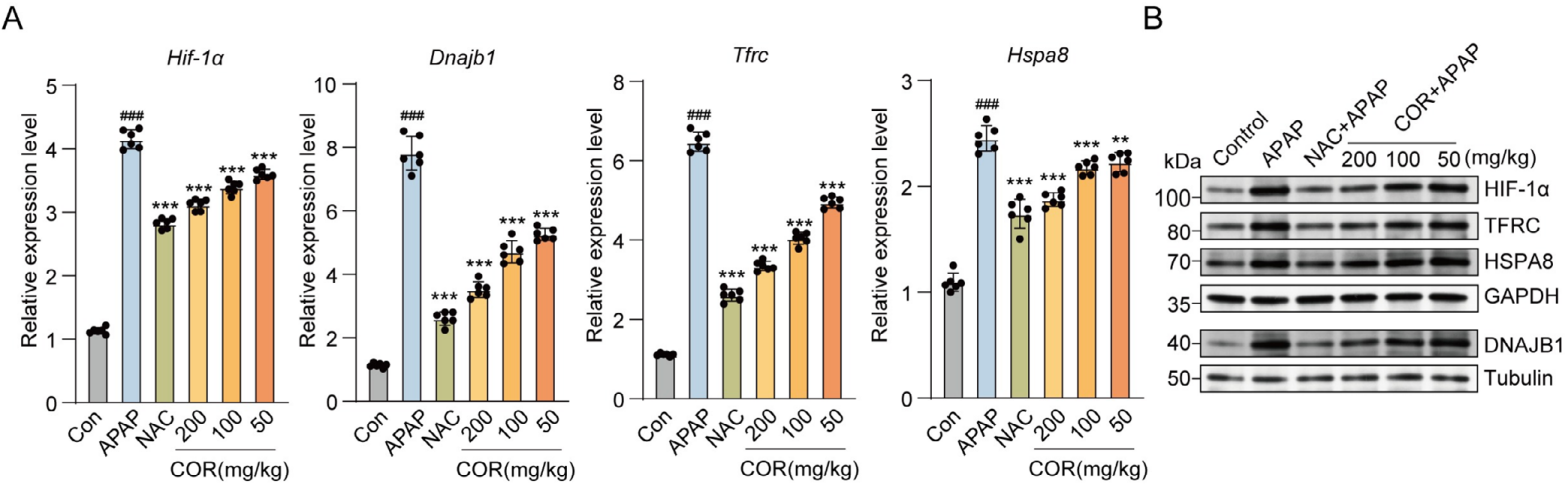
qRT‐PCR and Western blot validation of key genes identified by RNA‐seq. (A) Relative mRNA expression levels of *Hif‐1α*, *Dnajb1*, *Tfrc* and *Hspa8* in mouse liver tissues from the Con, APAP, NAC, and COR (50, 100, 200 mg/kg) + APAP groups. Data are presented as mean ± SD (*n* = 6). ###*p* < 0.001 vs. Con; **p < 0.01, ***p < 0.001 vs. APAP. (B) Immunoblot validation of key targets in liver lysates. Upper, HIF‐1α, TFRC and HSPA8 with GAPDH as a loading control; lower, DNAJB1 with Tubulin as a loading control. Samples include Control, APAP, NAC + APAP, and COR + APAP (50, 100, 200 mg/kg) groups.

To validate the reliability of our RNA‐seq data, we first performed qRT‐PCR on a subset of representative genes, including *Hif‐1α*, *Dnajb1*, *Tfrc*, and *Hspa8*. As shown in Figure 3A, APAP exposure significantly altered the expression of these genes. Notably, COR pretreatment largely reversed or partially restored these transcriptional abnormalities.

Following this transcriptional validation, we next examined key molecules at the protein level. The Western blot results (Figure 3B) aligned well with the qRT‐PCR trends, confirming that the differential expression observed in the transcriptomic data translated into corresponding changes in protein abundance. Overall, the consistency between the mRNA and protein data strongly supports the credibility of our RNA‐seq findings. Because assessing only a small panel of selected proteins may introduce bias, we decided to analyze the activation of ATF6, which undergoes proteolytic cleavage; the released translocates to the nucleus and triggers the expression of numerous downstream ER stress‐related factors [48]. Reassuringly, we observed that APAP exposure significantly activated ATF6, whereas COR pretreatment effectively reversed this dysregulation (Figure S2). Taken together, our results suggest that COR protects against APAP‐induced liver injury by restoring proteostasis.

### 2.4 COR Suppresses APAP‐Induced Inflammatory Responses

Transcriptomic profiling suggested that COR treatment may dampen inflammatory activation; therefore, we further quantified representative inflammatory readouts for validation. Inflammation‐driven hepatotoxicity is a shared and critical pathogenic mechanism across diverse forms of liver injury, and APAP overdose represents a prototypical example in which inflammatory signaling amplifies the injury cascade and aggravates hepatocellular damage [38].

**FIGURE 4:**
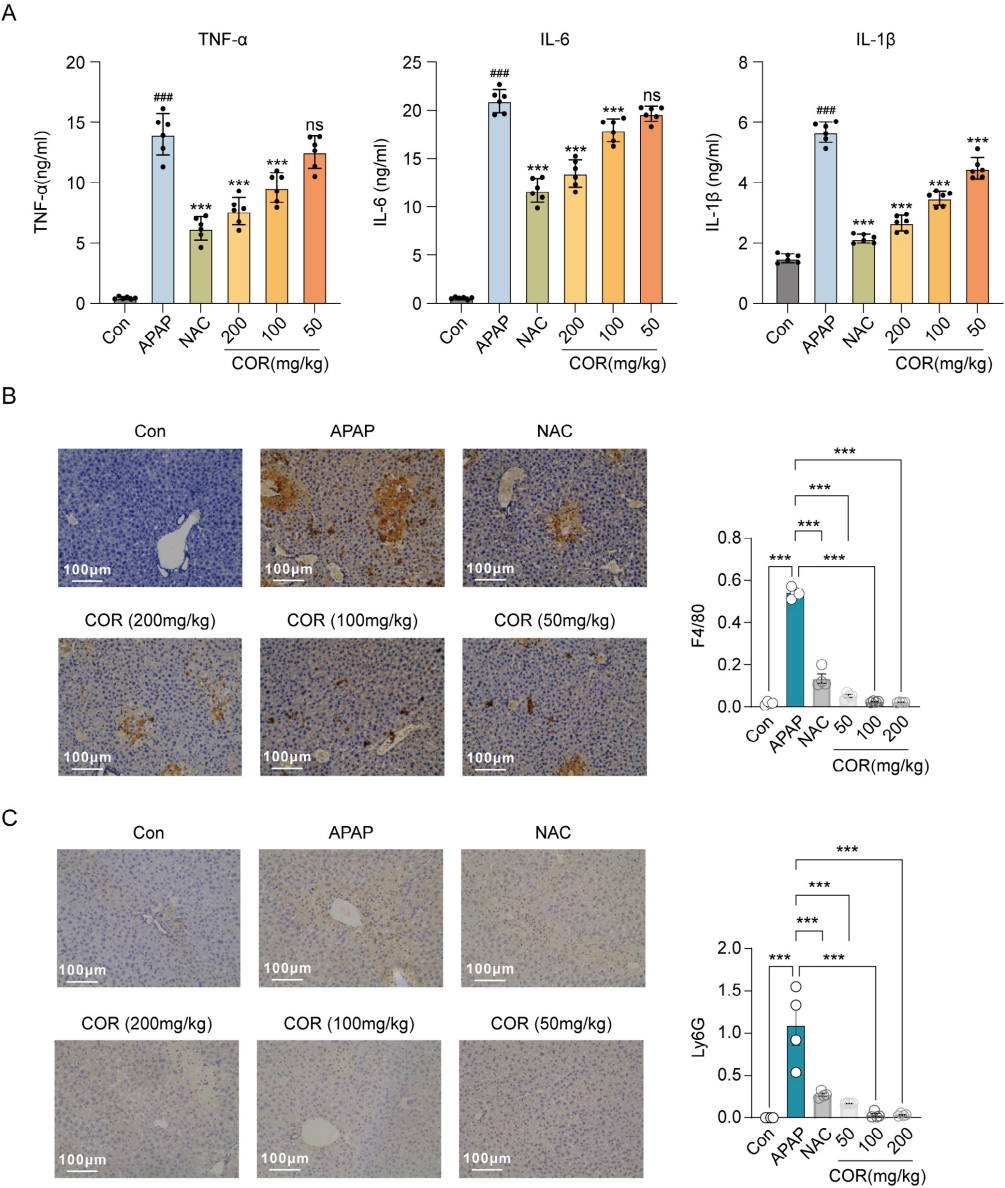
Cordycepin attenuates APAP‐induced inflammation. (A) Serum levels of pro‐inflammatory cytokines TNF‐α, IL‐6, and IL‐1β measured by ELISA in mice from the Con, APAP, NAC + APAP, and COR + APAP groups (COR 50, 100, and 200 mg/kg). Data are presented as mean ± SD (*n* = 6). ***p < 0.001, **p < 0.01, *p < 0.05 versus APAP group; ###*p* < 0.001 versus control group; ns, not significant. (B) Representative immunohistochemical staining of F4/80 in liver sections (left) and semi‐quantification of F4/80‐positive signals (right), indicating hepatic macrophage accumulation. Data are presented as mean ± SD (*n* = 4). Statistical analysis was performed using one‐way ANOVA followed by Tukey's multiple‐comparisons test. *p < 0.05, **p < 0.01, ***p < 0.001 (comparisons as indicated). (C) Representative immunohistochemical staining of Ly6G in liver sections (left) and semi‐quantification of Ly6G‐positive signals (right), indicating hepatic neutrophil infiltration. Data are presented as mean ± SD (*n* = 4). Statistical analysis was performed using one‐way ANOVA followed by Tukey's multiple‐comparisons test. *p < 0.05, **p < 0.01, ***p < 0.001 (comparisons as indicated).

To assess the magnitude of inflammatory responses, we measured serum concentrations of TNF‐α, IL‐6, and IL‐1β based on ELISA. The pathogenesis of acute liver injury is closely linked to a sequential inflammatory response, involving the early initiation by IL‐6, signal amplification by TNF‐α, and inflammasome‐dependent disease progression driven by IL‐1β [49, 50, 51]. We found that APAP exposure robustly caused hepatic inflammatory responses, as evidenced by marked elevations of serum TNF‐α, IL‐6, and IL‐1β, indicating that APAP exacerbates liver injury through activation of inflammatory pathways. In contrast, COR pretreatment—particularly at the higher dose (200 mg/kg)—significantly reduced these cytokine levels, demonstrating that COR effectively attenuates APAP‐triggered inflammation (Figure 4A).

Immunohistochemical analyses further corroborated these findings. In liver sections, APAP treatment markedly increased the number of F4/80^+^ cells, indicating prominent macrophage infiltration during APAP‐induced hepatic inflammation. COR pretreatment substantially decreased F4/80^+^ cell accumulation, suggesting that COR suppresses macrophage activation and recruitment (Figure 4B). In addition, Ly6G^+^ neutrophils were significantly increased following APAP challenge, underscoring the contribution of neutrophil infiltration to the inflammatory response. High‐dose COR markedly reduced Ly6G^+^ neutrophil accumulation, further supporting the role of COR in limiting inflammatory cell infiltration (Figure 4C). Collectively, these data indicate that COR mitigates APAP‐induced hepatic inflammation by restraining macrophage and neutrophil infiltration, highlighting its potential immunomodulatory contribution to hepatoprotection.

### 2.5 COR Alleviates APAP‐Induced Oxidative Stress and Cell Death

Given that the above results demonstrate a pronounced anti‐inflammatory function of COR in the APAP model, we further hypothesized that COR might concomitantly attenuate inflammation‐associated oxidative injury. To test this hypothesis, we quantified hepatic levels of the lipid peroxidation product MDA as well as the antioxidant defense indicators catalase (CAT), GSH, and SOD. MDA is widely used as a readout of lipid peroxidation [52]. GSH represents a major non‐enzymatic antioxidant component, whereas SOD and CAT contribute to the detoxification of superoxide anions, together forming a critical cellular defense barrier against ROS‐mediated injury [53].

**FIGURE 5:**
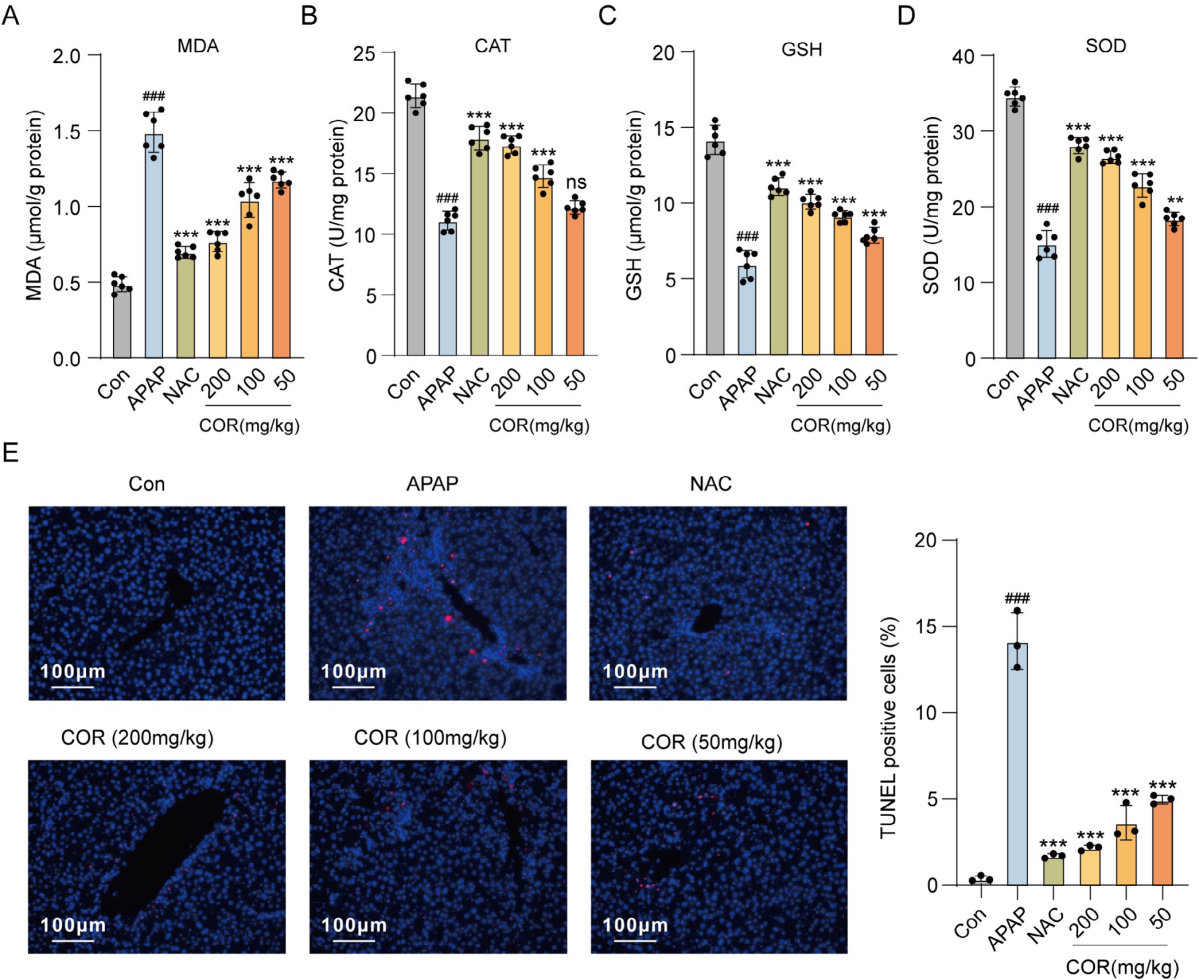
Cordycepin mitigates APAP‐induced oxidative stress and apoptosis. (A–D) Hepatic oxidative stress and antioxidant capacity were assessed by measuring (A) malondialdehyde (MDA; lipid peroxidation), (B) catalase (CAT) activity, (C) glutathione (GSH) content, and (D) superoxide dismutase (SOD) activity. Data are mean ± SD (*n* = 6). One‐way ANOVA with Tukey's multiple comparisons. ***p < 0.001, **p < 0.01, *p < 0.05 versus APAP group; ###*p* < 0.001 versus control group; ns, not significant. (E) Representative TUNEL staining of liver sections from the Control (Con), APAP, NAC (200 mg/kg), and COR (50, 100, 200 mg/kg) groups after APAP challenge (400 mg/kg). TUNEL‐positive nuclei are shown in red, and nuclei are counterstained with DAPI (blue). Scale bar, 100 μm. The bar graph shows the percentage of TUNEL‐positive cells in each group. Data are presented as mean ± SD (*n* = 3). Statistical analysis was performed using one‐way ANOVA followed by Tukey's multiple‐comparisons test. ###*p* < 0.001 vs. Con; ***p < 0.001 vs. APAP.

The results showed that APAP overdose markedly increased hepatic MDA levels, indicating pronounced oxidative stress injury (Figure 5A). In contrast, pretreatment with either NAC or COR effectively attenuated the APAP‐induced elevation of MDA, suggesting a reduced oxidative burden. Consistently, APAP challenge significantly decreased hepatic CAT activity as well as GSH and SOD levels, whereas NAC and high‐dose COR (200 mg/kg) largely restored these antioxidant indices (Figure 5B–D). Collectively, these findings indicate that COR pretreatment enhances hepatic antioxidant defenses and thereby alleviates APAP‐induced oxidative stress. Given that excessive inflammatory responses and oxidative stress can trigger hepatocyte apoptosis, and that COR treatment markedly alleviated inflammation and reduced oxidative injury, we hypothesized that COR might also protect hepatocytes from apoptosis induced by APAP overdose. Subsequently, we performed TUNEL staining. As expected, APAP challenge resulted in extensive TUNEL‐positive cells, indicating prominent hepatocyte apoptosis, whereas COR pretreatment significantly reduced apoptotic signals, demonstrating a clear anti‐apoptotic effect (Figure 5E). Overall, our results suggest that COR may protect against APAP‐caused drug‐induced liver injury by enhancing hepatic antioxidant defenses, reducing oxidative stress burden, and inhibiting hepatocyte apoptosis.

## 3 Discussion

Natural products from herbal medicines have long served as a rich source of drug leads, particularly for complex conditions where multi‐pathway regulation is desirable [54, 55, 56]. In this context, “medicine–food homology” (MFH) materials—defined as botanicals (or related natural resources) that are traditionally consumed as foods with an established record of safety may offer practical translational advantages [2, 9]. Notably, MFH materials are typically expected to have a long history of dietary consumption and to pass safety assessment before being widely adopted as functional ingredients.

*C. militaris* , a representative medicinal–edible fungus, is widely used in Asia and contains multiple bioactive constituents, among which COR is a hallmark nucleoside component [14, 57]. Experimental profiling of *C. militaris* ‐derived preparations confirms that cordycepin can be detected and quantified alongside adenosine, supporting its feasibility as a source‐derived intervention molecule [19]. From a safety standpoint, previous research found that COR is tolerated within reasonable dose ranges in preclinical settings, with genotoxicity readouts (including Ames‐type testing) reported as weak or negative in relevant evaluations—features that collectively encourage further exploration of its use as a supportive hepatoprotective candidate rather than a single‐target replacement drug [58]. Our in vivo data show that COR pretreatment is associated with an overall hepatoprotective trend in the APAP model. Together, these observations support the idea that bioactive constituents originating from well‐defined MFH resources may be particularly suitable for adjunctive strategies aimed at buffering inflammation/oxidative stress–driven injury cascades; nevertheless, rigorous dose–exposure–response characterization and longer‐term safety‐window assessments remain necessary for clinical translation.

Given these translational considerations, a practical next question is which high‐burden liver‐injury settings may most plausibly benefit from an MFH‐derived, multi‐target supportive candidate. Among drug‐induced liver injury etiologies, APAP overdose represents a prototypical dose‐dependent acute liver injury with rapid progression and well‐characterized pathogenic cascades, while the clinical benefit of the first‐line antidote N‐acetylcysteine (NAC) remains highly time‐ and protocol‐dependent [33, 34]. Importantly, APAP hepatotoxicity is driven not only by NAPQI‐associated oxidative stress and mitochondrial dysfunction, but also by inflammatory amplification, ER stress imbalance, and downstream cell‐death outcomes [28, 29, 30, 31, 32]. Therefore, COR—given its reported anti‐inflammatory and antioxidant activities and its potential to buffer stress‐response programs—appears conceptually aligned with a multi‐pathway “adjunctive hepatoprotection” profile, providing a clear rationale to interrogate its effects and mechanisms in the APAP model.

To define the molecular basis of COR‐mediated protection, we performed transcriptomic profiling and focused on candidate genes that were significantly dysregulated following APAP exposure but displayed a “reversal/normalization” trend upon COR intervention. Such genes provide an unbiased framework to identify key regulatory nodes and pathway architectures through which COR may counteract APAP hepatotoxicity. KEGG enrichment highlighted coordinated alterations in protein processing in the ER and the HIF‐1 signaling pathway during APAP‐induced hepatotoxicity, with multiple changes being substantially attenuated by COR. Within the ER proteostasis network, HSPA8 and DNAJB1 were markedly induced after APAP treatment, suggesting an increased protein quality‐control burden; COR reduced their abnormal upregulation, implying that COR may alleviate proteostatic stress and improve stress‐related outcomes. Similarly, in the HIF‐1 pathway, HIF1A and related nodes (e.g., TFRC) exhibited a consistent “APAP upregulation–COR reversal” pattern, supporting the notion that APAP‐triggered oxidative stress and hypoxia‐like transcriptional adaptation may contribute to injury amplification, whereas COR may partially restrain overactivation of this stress‐response axis. The spinocerebellar ataxia enrichment signal was largely driven by proteasome components (e.g., *Psmd4*, *Psmc2*, *Adrm1*, *Psmd7*), autophagy‐related factors (*Rb1cc1*, *Wipi2*), and mitochondrial quality‐control genes (*Afg3l2*). Thus, this enrichment more likely reflects activation of the UPS (ubiquitin‐proteasome system)–autophagy–mitochondrial stress network in APAP hepatotoxicity, rather than indicating a cerebellar‐specific process. Thus, the transcriptomic data suggest that COR‐mediated hepatoprotection involves coordinated regulation across multiple pathways, spanning suppression of inflammation, attenuation of oxidative stress, and improvement of cell‐death outcomes. Guided by these findings, we conducted independent validation experiments to assess inflammatory status, oxidative stress levels, antioxidant defense. These results were consistent with the transcriptomic signatures and support a mechanism whereby COR improves liver function and histological damage primarily through reducing inflammatory burden and oxidative injury.

Taken together, our study demonstrates that COR significantly alleviates APAP‐induced liver damage. This effect is attributable to coordinated modulation of ER proteostasis, HIF‐1–associated stress responses, and the proteasome–autophagy–mitochondrial quality‐control network, thereby conferring anti‐inflammatory and antioxidant effects and improving cell‐death outcomes. Notably, advancements in synthetic biology technologies have enabled the identification of the biosynthetic gene clusters responsible for COR production [57, 59, 60, 61]. It is feasible to envision the large‐scale production of COR in chassis organisms in the near future.

While our study demonstrates the hepatoprotective mechanisms of COR, it is important to acknowledge the limitations of the prophylactic treatment model employed. In real‐world clinical settings, APAP hepatotoxicity typically presents as an acute emergency following an overdose, requiring rapid post‐exposure intervention. We believe this represents a unique advantage of COR from *C. militaris* . As this edible fungus is widely consumed as a food ingredient, it holds considerable potential for use in dietary intervention strategies. Accordingly, it is reasonable to propose its application as a functional food or dietary supplement for the proactive enhancement of hepatic defense mechanisms in high‐risk populations—for instance, individuals undergoing long‐term pharmacotherapy or those with underlying metabolic disorders—representing a promising translational avenue. Nonetheless, rigorous validation through well‐designed clinical trials is essential to verify its efficacy. Another limitation of this study is that only male mice were employed. Although this represents a standard experimental practice in the field [36, 37], future investigations should include female mice, which may yield important new insights to support the development of active agents against drug‐induced liver injury.

## 4 Materials and Methods

### 4.1 Reagents

APAP (purity: 99%; A105808) was bought from Aldrich (MO, USA). NAC (purity: 99%; 616‐91‐1) and COR (purity: 97%; 73‐03‐0) were obtained from Med Chem Express (NJ, USA). APAP was dissolved in pre‐warmed saline (66°C) immediately before use, and COR was dissolved in H_2_O. Unless noted otherwise, all other reagents were bought from Sigma‐Aldrich.

### 4.2 Animal Experiments

Male C57BL/6 mice were bought from Vital River Company. All experimental protocols were approved by the Committee and performed in compliance with applicable animal welfare regulations. Mice were maintained under controlled conditions (25°C ± 1°C, 60% ± 5% RH) and allowed to acclimate for 7 days before experimentation. Following acclimation, animals were randomly assigned to six groups: Control, NAC + APAP (NAC 200 mg/kg), APAP (400 mg/kg), and COR + APAP at three doses (COR 50, 100, or 200 mg/kg). For pretreatment, NAC (200 mg/kg; dissolved in water) or COR (50/100/200 mg/kg) was administered once daily by oral gavage for 7 consecutive days, whereas mice in the control and APAP groups received an equal volume of saline. Three hours after the final pretreatment, mice in all groups except the control group were challenged with APAP (400 mg/kg, intragastric). At 12 h post‐APAP administration, animals were killed; blood was gathered via orbital sampling, while liver tissues were excised for downstream experiment.

### 4.3 Biochemical and ELISA Assays

Plasma ALT and AST activities were detected via assay kits following the manufacturers' instructions. For hepatic oxidative‐stress assessment, liver tissues were homogenized on ice and analyzed using assay kits for SOD, CAT, and GSH and for MDA (Beyotime, Shanghai, China). Serum levels of TNF‐α, IL‐6, and IL‐1β were detected through ELISA kits (Multi Sciences, Hangzhou, China).

### 4.4 RT–qPCR Analysis

Total RNA was isolated with TRIzol reagent. Complementary DNA (cDNA) was generated from purified RNA via reverse transcription kit. And qPCR was used with SYBR Green Master Mix (Takara, Shiga, Japan) on PCR platform. Primer sequences are provided in Table S1.

### 4.5 Immunoblot Analysis

Tissue samples were homogenized and lysed in RIPA buffer containing inhibitor cocktails (MA, USA). Total protein contents were quantified on BCA assay kit. Equivalent amounts of protein were resolved and then transferred onto PVDF membranes (Merck, MA, USA). Membranes were blocked in 5% (w/v) non‐fat milk and incubated overnight at 4°C with the indicated primary antibodies: TUBULIN and GAPDH (Proteintech, Wuhan, China), TFRC, HSPA8, and HIF‐1α (Cell Signaling Technology, MA, USA), and DNAJB1 (Proteintech, Wuhan, China). After washing, membranes were incubated with HRP‐labeled secondary antibodies (1:8000) for 1 h. Immunoreactive bands were visualized through ECL reagent (Biosharp, Beijing, China) and recorded, then quantified through ImageJ software and normalized with GAPDH or TUBULIN as indicated.

### 4.6 Histopathological Analysis and Immunohistochemistry

Liver tissue sections were used to evaluate liver structure, cell death, and inflammatory cell infiltration by H&E, TUNEL staining, and immunohistochemistry, respectively. The liver samples were first fixed in formalin, then cut into 5 μm sections. Before staining, the sections were rehydrated through a graded ethanol series. TUNEL staining was carried out with a commercial kit following the instructions. For immunohistochemistry, antigen retrieval was conducted by heat treatment in sodium citrate buffer. Subsequently, it was incubated for 12 h at 4°C with primary antibodies against F4/80 or Ly6G, followed by a biotinylated secondary antibody. The signal was developed with DAB (Gene Tech), and the nuclei were counterstained. Images were taken via a Leica DMi8 microscope (Wetzlar, Germany). For each section, at least three random fields were selected and analyzed with ImageJ.

### 4.7 Transcriptomic Sequencing and Data Processing

Liver samples were sent from nine mice to Novogene (Beijing, China) for RNA sequencing. In brief, total RNA was extracted from the samples in the Control, APAP, and COR + APAP (COR 200 mg/kg) groups using TRIzol, and cDNA libraries were then prepared and sequenced on an Illumina platform. Sequencing reads were aligned to obtain mapping results. Differentially expressed genes (DEGs) were called with DESeq2 using adjust *p* < 0.05 and fold change > 1.5 as the cutoff criteria. KEGG pathway enrichment was performed using the online Metascape tool (https://metascape.org) [62].

### 4.8 Statistical Analysis

All data were analyzed in a blinded manner, and the analyst was unaware of group allocation prior to statistical testing. Data were depicted as mean ± SD. GraphPad Prism 10 (Edition 6, USA) was applied to treat the data. Statistical testing was conducted when the sample size in each group was ≥ 3. Differences among groups were evaluated by ANOVA and the relevant post hoc test. The significance level was set as 0.05.

## 5 Conclusions

COR pretreatment protects against APAP‐induced liver injury. In our model, it was associated with lower ALT/AST and milder histological damage. This was accompanied by reduced inflammatory signals, improved antioxidant status (less lipid peroxidation), and fewer apoptotic cells. The RNA‐seq results also point to changes in stress‐related pathways, including ER protein processing and HIF‐1/PPAR signaling, which may help explain the overall protective trend.

## Author Contributions

N.L. conceived and guided this project. C.F. carried out most of the animal experiments. S.L. assisted with the animal experiments and data analysis. S.S. contributed to sample collection and method development. C.X., J.L., and N.L. supervised the project and interpreted the results. C.F., S.L., and N.L., wrote the manuscript with input from the other authors. All authors read and approved the final manuscript.

## Funding

This research was funded by the Major Science and Technology Innovation Project of China Academy of Chinese Medical Sciences (CI2024E003KC‐07), and the Fundamental Research Funds for the Central Public Welfare Research Institutes (GLQH2025010).

## Ethics Statement

All animal procedures were reviewed and approved by the Institutional Animal Care and Use Committee of the China Academy of Chinese Medical Sciences (IBTCMCACMS‐2503009).

## Consent

The authors have nothing to report.

## Conflicts of Interest

The authors declare no conflicts of interest.

## Supporting information


**Figure S1:** Transcriptomic profiling and differential expression analysis in Con, APAP, and COR + APAP groups. (A) KEGG pathway enrichment of DEGs between the Con and APAP groups (corresponding to the DEGs shown in the volcano plot described in Figure 2C). The bubble plot displays the RichFactor (x‐axis), enriched pathways (y‐axis), gene number (bubble size), and q‐value (bubble color). (B) KEGG pathway enrichment of DEGs between the APAP and COR + APAP groups (corresponding to the DEGs shown in the volcano plot described in Figure 2D). The bar plot shows the top enriched pathways ranked by −log_10_(*P*).
**Figure S2:** Representative Western blot images showing the protein expression of ATF6 in the Control, APAP, NAC + APAP, and COR + APAP (50, 100, and 200 mg/kg) groups. GAPDH was utilized as an internal loading control.

**Table S1:** Primer used in this study.

**Table S2:** The count, fpkm matrix and DEGs from RNA‐seq.

## Data Availability

The data that supports the findings of this study are available in the Supporting Information of this article.
