# Supplementary material for: Edible Fungus Compound Cordycepin Protects Against Acetaminophen‐Induced Liver Injury
Source: J Clin Lab Anal. 2026 May 25;40(11):e70266. doi: 10.1002/jcla.70266 (PMC13267160; doi:10.1002/jcla.70266)
Supplement: Supplementary file 1 — Figure S1: Transcriptomic profiling and differential expression analysis in Con, APAP, and COR + APAP groups. (A) KEGG pathway enrichment of DEGs between the Con and APAP groups (corresponding to the DEGs shown in the volcano plot described in Figure 2C). The bubble plot displays the RichFactor (x‐axis), enriched pathways (y‐axis), gene number (bubble size), and q‐value (bubble color). (B) KEGG pathway enrichment of DEGs between the APAP and COR + APAP groups (corresponding to the DEGs shown in the volcano plot described in Figure 2D). The bar plot shows the top enriched pathways ranked by −log10(P). Figure S2: Representative Western blot images showing the protein expression of ATF6 in the Control, APAP, NAC + APAP, and COR + APAP (50, 100, and 200 mg/kg) groups. GAPDH was utilized as an internal loading control. [file JCLA-40-e70266-s003.docx]

**Supplementary material**

**Edible fungus compound cordycepin protects against acetaminophen-induced liver injury**

Chunjin Fu^1,^ ^†^, Shuyu Li^1,^ ^†^, Shengnan Shen^1^, Chengchao Xu^1^, Jingjing Liao^1, *^, Na Lin^1, *^

^1^State Key Laboratory for Quality Assurance and Sustainable Use of Dao-di Herbs, China Academy of Chinese Medical Sciences, Beijing 100700, China

^†^These authors contributed equally

^*^Corresponding authors: [jjliao@icmm.ac.cn](mailto:jjliao@icmm.ac.cn) (J.L.), [linna888@163.com](mailto:linna888@163.com) (N.L.)


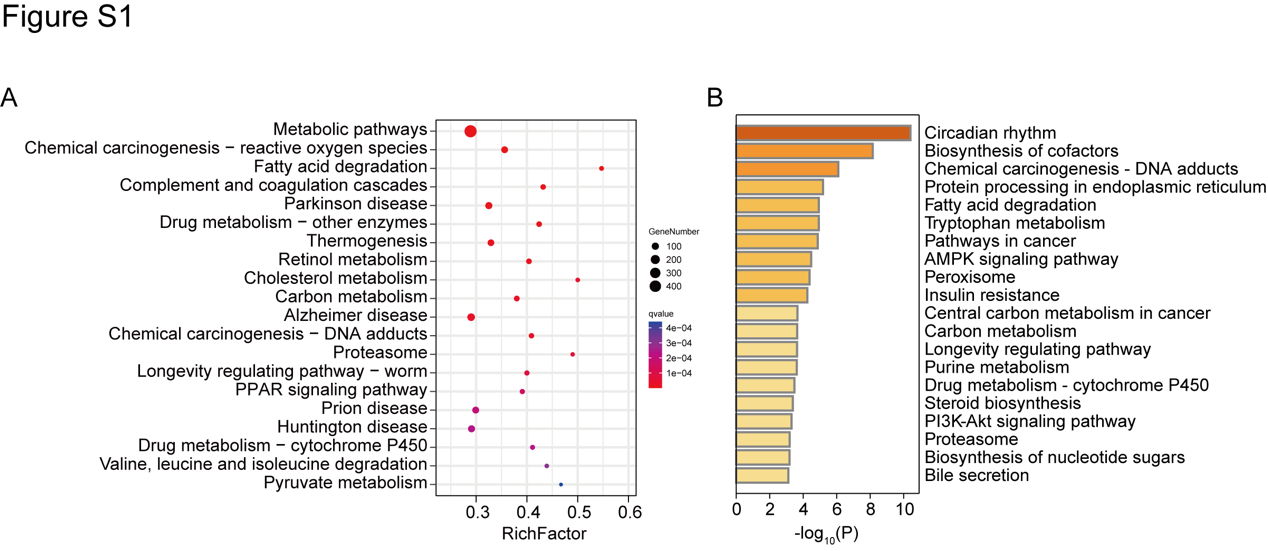


**Figure S1.** Transcriptomic profiling and differential expression analysis in Con, APAP, and COR+APAP groups. **(A)** KEGG pathway enrichment of DEGs between the Con and APAP groups (corresponding to the DEGs shown in the volcano plot described in Figure 2C). The bubble plot displays the RichFactor (x-axis), enriched pathways (y-axis), gene number (bubble size), and q-value (bubble color). **(B)** KEGG pathway enrichment of DEGs between the APAP and COR+APAP groups (corresponding to the DEGs shown in the volcano plot described in Figure 2D). The bar plot shows the top enriched pathways ranked by −log_10_(*P*).


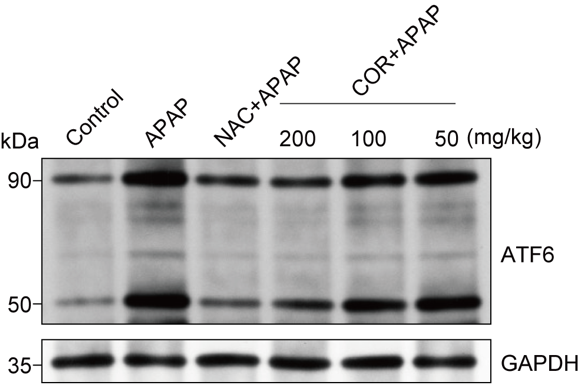


**Figure S2.** Representative Western blot images showing the protein expression of ATF6 in the Control, APAP, NAC+APAP, and COR+APAP (50, 100, and 200 mg/kg) groups. GAPDH was utilized as an internal loading control.

**Table S1. Primer used in this study.**

“Table S1.xlsx”

**Table S2. The count, fpkm matrix and DEGs from RNA-seq.**

“Table S2.xlsx”
